# Standardised Methods for Developing Conceptual Frameworks for Placental Disorders of Pregnancy: Pre‐Eclampsia and Stillbirth

**DOI:** 10.1111/1471-0528.18083

**Published:** 2025-02-19

**Authors:** Terteel Elawad, Mai‐Lei Woo Kinshella, Ellie Stokes, Kelly Pickerill, Elisa Dalle Piagge, Marianne Vidler, Ella Stanley, Marie‐Laure Volvert, Jeffrey N. Bone, Helen Elwell, Hiten D. Mistry, Violet Mateljan, Eleni Tsigas, Veronique Filippi, Peter von Dadelszen, Hannah Blencowe, Laura A. Magee, PRECISE Network

**Affiliations:** ^1^ Royal Free London NHS Foundation Trust London UK; ^2^ Department of Obstetrics and Gynaecology BC Children's and Women's Hospital and University of British Columbia Vancouver British Columbia Canada; ^3^ Royal United Hospitals Bath NHS Foundation Trust Bath UK; ^4^ School of Midwifery University of British Columbia Vancouver British Columbia Canada; ^5^ NHS Lothian Foundation School Edinburgh UK; ^6^ School of Medicine University of Leeds Leeds UK; ^7^ BMA Library British Medical Association London UK; ^8^ Department of Women and Children's Health King's College London London UK; ^9^ Department of Population Health Sciences, College of Life Sciences University of Leicester Leicester UK; ^10^ Preeclampsia Foundation Canada Fonthill Ontario Canada; ^11^ Preeclampsia Foundation Melbourne Florida USA; ^12^ Centre for Maternal Adolescent Reproductive and Child Health (MARCH), London School of Hygiene and Tropical Medicine London UK

**Keywords:** conceptual framework, fetal growth restriction, placental disorders, pre‐eclampsia, preterm birth, stillbirth

## Abstract

**Background:**

Risk factors for the placental disorders of pregnancy (pre‐eclampsia, fetal growth restriction, preterm birth, and stillbirth) are complex, frequently involving the interplay between clinical factors and wider social and environmental determinants of health. Biomarkers modulate the maternal and fetal responses to biological processes that underlie the development of placental disorders.

**Objectives:**

To develop a standardised methodology to assess the importance of, and inter‐relationships between, candidate risk factors for the various placental disorders.

**Search Strategy:**

Systematic searches were conducted using Medline, Embase, Health Technology Assessments, Database of Abstracts of Reviews of Effects, Cochrane Library databases, Google Scholar, and reference lists of retrieved papers.

**Selection Criteria:**

We deployed a hierarchy of reviews, systematic reviews, and cohort studies with at least 1000 participants (100 for biomarker studies), published in the prior decade.

**Data Collection and Analysis:**

We assessed the strengths of association and quality of evidence linking risk factors with individual placental outcomes.

**Conclusions:**

We have developed a standardised approach to assess the importance and inter‐relatedness of putative risk factors for the placental disorders of pregnancy.

## 1 Introduction

The placental disorders of pregnancy, including pre‐eclampsia, fetal growth restriction, preterm birth, stillbirth and others, have complex risk factors reported across vast literature landscapes, often fragmented by discipline. While clinical practice guidelines (CPGs), review articles, and textbooks frequently list risk factors for each of the placental disorders [1, 2], many of those lists have been carried forward from previous CPGs and reviews, or studies that have been superseded. Most CPGs and review articles solely describe clinical risk factors that can be assessed at the time of booking for antenatal care or during the first trimester (e.g., SPREE model to identify those women who would benefit from aspirin to reduce the risk of preterm pre‐eclampsia [3]), and do not include additional risks that arise later in pregnancy (e.g., gestational diabetes as a risk for pre‐eclampsia). Previously, Hiatt et al. described a methodology for stratifying levels of evidence to develop a comprehensive conceptual framework model of determinants of postmenopausal breast cancer [4]. Using an evolution of the Hiatt methodology, we identified that many clinical risk factors listed in pregnancy hypertension CPGs were not well aligned with current evidence [5].

As a consortium, we have formed the PRECISE (Pregnancy Care Integrating translational Science, Everywhere) Network, that has recruited ≈6900 unselected pregnant women at the time of booking for antenatal care (and, for comparison, ≈1200 non‐pregnant women of reproductive age). PRECISE has gathered social and clinical data, and an associated biorepository, to understand the complex pathways to optimal and complicated pregnancy outcomes in three sub‐Saharan African countries: The Gambia (West Africa), Kenya (East Africa), and Mozambique (Southern Africa) [6]. Pre‐eclampsia, fetal growth restriction, preterm birth and stillbirth complicate up to a third of pregnancies in sub‐Saharan Africa, and are associated with a global burden of approximately 46 000 maternal, two million fetal and newborn deaths annually, with a far greater burden of survived morbidity [7, 8, 9, 10, 11, 12].

As an organising principle, our objective was to further develop the standardised approach of Hiatt et al., to assess the strength of association and quality of evidence linking social, clinical, and biomarker risk factors for placental disorders globally, and the inter‐relatedness between the strongest factors. In this Supplement, using that methodology, we focus on pre‐eclampsia and stillbirth.

## 2 Methods

We modified the methods of Hiatt et al. to develop a comprehensive approach to model the determinants of the placental disorders [4]. Our approach involved convening expert groups, patient‐partner engagement, a hierarchical literature review, and assessing association strength and certainty of evidence using GRADE (Grading of Recommendations, Assessment, Development, and Evaluation).

### 2.1 Consultations With Field Experts

A broad group of experts in these pregnancy disorders was assembled from the Epidemiology, Social Determinants, and Biological Working Groups of the PRECISE Network [13] to build an initial working model of known determinants. Hiatt et al. had four quadrants of focus (social‐cultural, behavioural, physical, and biological) [4], which informed our initial searches to refine the working model. In addition, we evaluated biomarkers for each placental disorders as measurable indicators of underlying biological states and processes. All discussions were informed by ongoing interactions with the relevant condition‐specific advocacy groups (e.g., Preeclampsia Foundation, Action on Pre‐eclampsia (APEC), International Stillbirth Alliance (ISA)).

### 2.2 Patient‐Partner Engagement

Our conceptual framework approach was further informed by patient partner engagement through the “Pathways to preeclampsia: A partnered approach to educational materials and knowledge translation” initiative with the Preeclampsia Foundation and the Preeclampsia Foundation Canada. Patient partners provide invaluable input by guiding and informing research activities through their lived experience, and ensuring the research activities are relevant, representative, and meaningful to patient and public audiences. Patient partners included former patients, survivors, and condition experts, and were invited through patient and community networks at the REACH BC Registry and the Preeclampsia Foundation. Six patient partners were selected based on availability and to ensure diversity of perspectives (ethnic, regional, occupational, age), and were compensated for their time. We convened seven meetings with patient partners between January and September 2022. Meetings included reviewing the basics of research and patient‐oriented research, discussing the project, identifying group goals and objectives, and reviewing existing evidence on the causal pathways and attributable risk in the development of pre‐eclampsia.

Based on engagement with patient partners, we revised the four quadrants used by Hiatt et al. [4] into three areas of focus for the literature searches: medical histories, social determinants and biomarkers. Nutrition was also highlighted by patient partners and within the Preeclampsia Registry as an area of interest [14, 15], which was further explored as part of social determinants [16].

### 2.3 Literature Search

The search strategy for each placental disorder was developed in consultation with a clinical librarian (HE) at the British Medical Association. Searches were undertaken using combination of terms for the placental disorder and potential determinants based on consultation with field experts in PRECISE working groups, preliminary scoping literature searches, and existing frameworks. Medline (Ovid), Embase and Evidence‐Based Medicine Reviews, which includes the Health Technology Assessments, Database of Abstracts of Reviews of Effects and Cochrane Library databases, Google Scholar, and reference lists were searched, using Medical subject heading (MESH) and free text words.

### 2.4 Seven‐Stage Hierarchical Approach to Data Extraction

**FIGURE 1:**
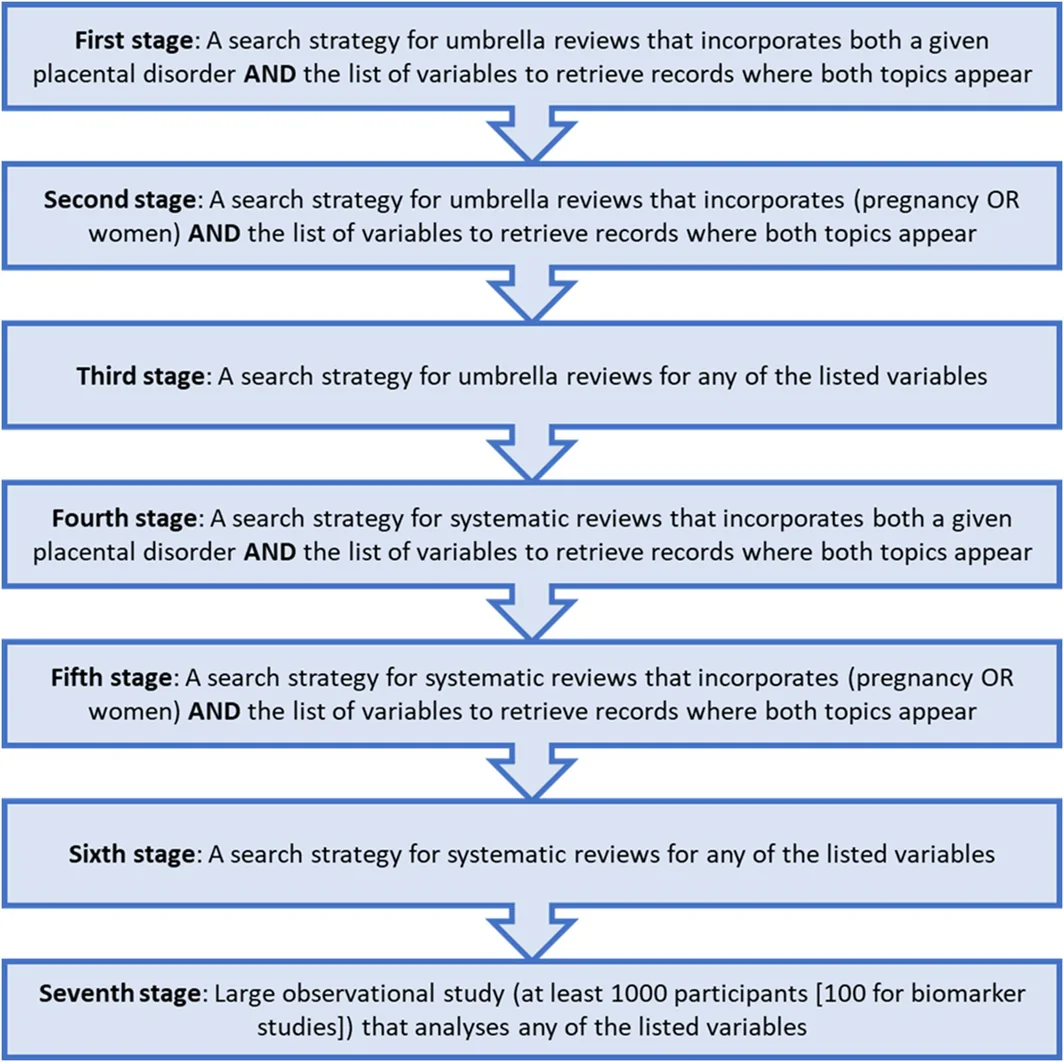
Hierarchical approach to data extraction.

With the aim of understanding a broad landscape of research, a hierarchical approach was utilised to identify the highest level of evidence supporting a relationship between a risk factor and a given placental disorder (Figure 1). The approach was purposefully designed to review large bodies of literature, pragmatically accommodating multiple study designs and prioritising publications with stronger evidence first. Umbrella reviews (reviews of systematic reviews) focused on the placental disorder in question were first sought. If no relevant umbrella reviews were identified, then the process was repeated to identify relevant systematic reviews, prioritising the most recent, highest quality review. If no systematic reviews were identified for the risk factor of interest, then large observational cohort studies (including secondary analyses of trials) were sought, searching individually for relevant risk factors. Observational studies with at least 1000 participants were targeted, as described by Bartsch et al. [17], to be more representative of the general population and to have sufficient statistical power to assess less prevalent, but potentially important, risk factors [18]. Given sample size standards in biomarker studies, this threshold was lowered to at least 100 participants for studies of biomarkers for pragmatic reasons.

Smaller observational studies with fewer than 1000 participants (100 for biomarker studies), cross‐sectional surveys, case‐controlled studies, case reports/series, qualitative reviews, and editorials were not considered.

### 2.5 Data Extraction

Titles and abstracts of articles were screened to assess eligibility. Potentially eligible studies underwent full‐text review. Data abstracted were general study characteristics, the characteristics necessary to assess study quality, and the strength of association between each risk factor and the specific placental disorder (estimated as relative risk (RR), odds ratio (OR) or diagnostic OR (DOR), and reported, adjusted where possible, or calculated from the prevalence of the placental disorder among women with and without the risk factor). In addition, subcategories of a potential risk factor were considered (e.g., body mass index [BMI] categorised as overweight or obese).

The strength of association between risk factors and the placental disorder of interest (e.g., stillbirth) was evaluated as definite, probable, possible, or not significant (Table 1) [19]. The evaluation was based on point estimates, extracted as reported or calculated from primary data using previously published thresholds [4, 20]. When studies reported outcomes as proportions, a risk ratio (RR) was calculated as a simple ratio between those with the risk factor of interest and those without. In addition, the results of the I^2^ statistic were extracted (or calculated from the Q statistic) to reflect heterogeneity. RRs and odds ratios (OR) were used interchangeably, as ORs are a reasonable approximation of the RR when the outcome occurs in less than 10% of the exposed and unexposed populations [21].

**TABLE 1:** Strength of association between risk factors and preeclampsia based on point estimates of various summary measures.

							Quality of evidence							
							*Iniital*	High		Moderate		Low		Very low
								Umbrella review or systematic review		—		Observational study (*N* > 1000)		—
							Evaluation/scoring	Risk of bias	Inconsis‐tency^c^	Indirect‐ness^c^	Impreci‐sion^c^	Publication bias^c^	Magni‐tude of effect^c^	Dose‐gradient response
								1↓ Lack of inclusion or discussion of sensi‐tivity analysis AND/OR 1↓ Study limitations	1↓ I^2^ > 50%	Excludes women from population 1↓ serious: 2↓ Very serious	1↓ Sample size < 1000 or not reported AND/OR 1↓ CI crosses 1.0	1↓ Asymmetrical funnel plots or no mention of public‐cation bias AND 1↓ evidence of very strong publication bias	1↑ Large: RR > 2–5 or 0.5–0.2 OR 2↑ Very large: RR > 5 or < 0.2	1↑ if existent
							*Final*	**High**		**Moderate**		**Low**		**Very low**
**Strength of association**		**RR or OR** ^a^		**DOR** ^**b**^	**LR**									
		(↑risk)	(↓ risk)		LR+	LR—								
	**Definite**	≥ 3.00	< 0.33	≥ 100	> 10	< 0.1								
	**Probable**	1.50–2.99	0.33–0.67	> 25–< 100	5.01–10.0	0.10–0.19								
	**Possible**	1.10–1.49	> 0.67– < 0.9	> 4–≤ 25	2.01–5.0	0.20–0.50								
	**Not significant**	0.90 to 1.09		1–4	1.0–2.0	0.51–0.99								

Abbreviations: DOR, diagnostic odds ratio; LR, likelihood ratio; LR‐, negative LR; LR+, positive LR; NS, not significant; OR, odds ratio; RR, relative risk.

^a^ Based on Hiatt and modification of Harvard Cancer Risk Index.

^b^ Based on LR+ and LR‐ criteria and definition of DOR as LR+/LR−.

^c^ Inconsistency was defined as variation between studies (heterogeneity), indirectness whether the paper answered the question we aimed to answer; imprecision defined according to the confidence interval of the summary estimates, publication bias as a tendency towards publication of studies that showed positive results, and magnitude of effect as determined by the RR.

### 2.6 Certainty of the Evidence

Quality of the evidence was rated independently by two reviewers from our multi‐disciplinary team [22] (Table 1). Following GRADE procedures, umbrella or systematic reviews that found an effect across a number of studies were considered to be higher certainty of evidence, while single observational studies were considered low certainty of evidence. Certainty of evidence could be upgraded for large effect sizes or evidence of a dose–response [23]. Certainty of the evidence could also be downgraded for risk of bias, inconsistency (substantial heterogeneity; I^2^ > 50%), indirectness (general populations without results specifically for pregnant populations), imprecision (wide confidence intervals), and publication bias (funnel plot asymmetry). Directness and precision were supported by our eligibility criteria, which excluded studies not conducted with pregnant populations and/or with small sample sizes.

### 2.7 Condensing the Frameworks

The approach described above results in a group of inter‐related frameworks focussed on social, clinical, and biomarker factors for each placental disorder. To build singular frameworks for each placental disorder, we next created a combined framework including all modifiable factors with strongest evidence (at least moderate strength of association and certainty of evidence) identified in the previous steps [4]. In this context, modifiability was defined as any determinant that could be modified through interventions at either individual or population level, namely, either altering societal norms, personal behaviours, or clinical pathways. Hence, modifiable determinants could be as varied as teenage pregnancy and educational level attained, through air quality, to blood pressure level during pregnancy and/or prescription of low‐dose aspirin.

## 3 Discussion

We have described a hierarchical approach to reviewing evidence, in combination with convening expert groups and engaging patient partners, to develop conceptual frameworks for the origins of pre‐eclampsia and stillbirth. This method is an evolution of that developed by Hiatt et al. used to develop a conceptual framework for the origins of postmenopausal breast cancer [4], adapted for the placental disorders of pregnancy and could be potentially extended to other maternity complications.

### 3.1 Strengths and Limitations

A key strength of this approach was the incorporation of patient perspectives. Our decision to merge behavioural factors into the medical histories and social determinant quadrants was in contrast to a separate behavioural quadrant in previous models [4], and was strongly motivated by our discussions with patient partners. Though pregnancy has been described as a “teachable moment” for weight and lifestyle interventions when women are motivated to have a positive maternity experience [24], research among women with pre‐eclampsia highlight the need for respectful counselling on the background and progression of the disease that adequately considers existing personal struggles and environmental constraints [15, 25, 26, 27]. Women with pre‐eclampsia have reported feelings of guilt, especially around stillbirths, the birth of small vulnerable newborns and potential impact over their children's future health [28, 29, 30, 31], and some have even reported feeling blamed by their care providers for their diagnosis [15]. Nesting behavioural factors into other quadrants highlights their clinical and social dimensions rather than focusing on individual choice.

Other main strengths of this approach is to further develop a published method to standardise the alignment of the strength of association between putative risk factors with placental outcomes, taking into account quality of evidence. In addition, this approach will inform analyses of modifiers and confounders of relationships between risks and outcomes. Furthermore, our explicit examination of biomarkers is valuable as biomarkers can play important role in detecting placental disorders early enabling interventions to improve outcomes.

The main limitation is the stepwise approach that prioritises umbrella reviews and systematic reviews over large cohort studies; a single well‐designed and adequately‐powered study may be better than combining poorer quality studies using mixed effects meta‐analytical approaches [32]. Our methodology of assessing certainty of evidence using GRADE and restricting inclusion to larger studies supports a high standard of evidence quality, but may presents challenges in representativeness. For example, large multi‐site assessments from tertiary hospitals may not necessarily represent underlying populations and the absence of robust cohort studies in resource‐limited settings may skew resulting conceptual frameworks towards findings from high‐income countries.

The final limitation is that this methodology paper does not describe the detailed assessment of the inter‐relatedness between risk factors identified through this approach. This limitation is addressed in this Supplement [33].

### 3.2 Interpretation

Currently, there are numerous clinical practice guidelines for each placental disorder, each of which has, in turn, numerous listed risk factors that have variable evidence to support them (e.g., pre‐eclampsia [34]). This results in clinical uncertainty due to the varied risk assessment approaches and implementation of interventions (e.g., risk assessment for preterm pre‐eclampsia and aspirin prescription [3]).

Previously, we piloted this approach to examine the evidence base for the lists of risk factors in the numerous pregnancy hypertension clinical practice guidelines [5, 34]. The Elawad et al. review excluded the biomarkers that are reviewed in this Supplement [35]. However, what we determined was a list of definite and probable risk factors that were aligned with the Fetal Medicine Foundation models that assess risk at 11–14 weeks and 35–36 week gestation [3, 36], other than exclusion of angiogenic factors that were not identified in the clinical practice guidelines.

An important implication of this approach, and the conceptual frameworks described in this Supplement, is that much of the obstetric literature is limited in terms of the size of informative cohort studies, especially qualitative and biomarker research, that caused them to be excluded from these analyses. That is not the case in cardiovascular and oncology research. As a research community, we need to take up this challenge to improve the quality of information upon which advice and care is based.

### 3.3 Conclusion

In conclusion, we recommend this standardised approach both to those building conceptual frameworks for these and other maternity disorders, and colleagues engaged in clinical practice guideline development. Although not without limitations, it would create an environment in which the recommendations provided to women and maternity care providers is standardised between jurisdictions, and provides support for adequately‐powered studies of optimal standard for inclusion as such frameworks evolve and mature.

## Author Contributions

Laura A Magee, Peter von Dadelszen, and Veronique Filippi conceptualised the conceptual framework and developed the methodology, with support from Terteel Elawad, Mai‐Lei Woo Kinshella, Ellie Stokes, Kelly Pickerill, Elisa Dalle Piagge, Marianne Vidler, Ella Stanley, Marie‐Laure Volvert, Jeffrey N. Bone, Helen Elwell, and Hiten D Mistry. Marianne Vidler, Violet Mateljan, and Eleni Tsigas led the patient engagement initiatives. Mai‐Lei Woo Kinshella wrote the first draft of the manuscript. All authors read and approved the final manuscript.

## Ethics Statement

The review only utilised data from previous published studies.

## Conflicts of Interest

The authors declare no conflicts of interest.

## Data Availability

Data sharing is not applicable to this article as no new data were created or analyzed in this study.
